# CAR Macrophages for SARS-CoV-2 Immunotherapy

**DOI:** 10.3389/fimmu.2021.669103

**Published:** 2021-07-23

**Authors:** Wenyan Fu, Changhai Lei, Zetong Ma, Kewen Qian, Tian Li, Jian Zhao, Shi Hu

**Affiliations:** ^1^ Department of Biophysics, College of Basic Medical Sciences, Second Military Medical University, Shanghai, China; ^2^ Team SMMU-China of the International Genetically Engineered Machine (iGEM) Competition, Department of Biophysics, Second Military Medical University, Shanghai, China; ^3^ Department of Assisted Reproduction, Shanghai Ninth People’s Hospital, Shanghai Jiao Tong University School of Medicine, Shanghai, China; ^4^ KOCHKOR Biotech, Inc., Shanghai, China

**Keywords:** SARS-CoV-2, COVID19, CAR, cell-based therapy, macrophages

## Abstract

Targeted therapeutics for the treatment of coronavirus disease 2019 (COVID-19), especially severe cases, are currently lacking. As macrophages have unique effector functions as a first-line defense against invading pathogens, we genetically armed human macrophages with chimeric antigen receptors (CARs) to reprogram their phagocytic activity against SARS-CoV-2. After investigation of CAR constructs with different intracellular receptor domains, we found that although cytosolic domains from MERTK (CAR_MERTK_) did not trigger antigen-specific cellular phagocytosis or killing effects, unlike those from MEGF10, FcRγ and CD3ζ did, these CARs all mediated similar SARS-CoV-2 clearance *in vitro*. Notably, we showed that CAR_MERTK_ macrophages reduced the virion load without upregulation of proinflammatory cytokine expression. These results suggest that CAR_MERTK_ drives an ‘immunologically silent’ scavenger effect in macrophages and pave the way for further investigation of CARs for the treatment of individuals with COVID-19, particularly those with severe cases at a high risk of hyperinflammation.

## Introduction

The coronavirus disease 2019 (COVID-19) pandemic has caused a sudden significant increase in hospitalizations for pneumonia with multiorgan disease and has led to more than 2,000,000 deaths worldwide. COVID-19 is caused by the novel severe acute respiratory syndrome coronavirus 2 (SARS-CoV-2), a novel enveloped RNA betacoronavirus. SARS-CoV-2 infection may be asymptomatic or cause a wide spectrum of symptoms, ranging from mild symptoms of upper respiratory tract infection to life-threatening sepsis (1). Manifestations of COVID-19 include asymptomatic carriers and fulminant disease characterized by sepsis and acute respiratory failure. Approximately 5% of patients with COVID-19, including 20% of those hospitalized, experience severe symptoms necessitating intensive care. More than 75% of patients hospitalized with COVID-19 require supplemental oxygen (1, 2). The case-fatality rate for COVID-19 varies markedly by age, ranging from 0.3 deaths per 1000 patients among patients aged 5 to 17 years to 304.9 deaths per 1000 patients among patients aged 85 years or older. Among patients hospitalized in the intensive care unit, the case fatality can reach 40% (1).

There are currently 102 SARS-CoV-2 vaccine candidates already under clinical evaluation and 185 in preclinical development (3). In the development of an effective vaccine, a number of challenges must be overcome, such as technical barriers, the feasibility of large-scale production and regulation, legal barriers, the potential duration of immunity and thus the number of vaccine doses needed to confer immunity, and the antibody-dependent enhancement effect. Moreover, there is another complicated area to consider: drug development for COVID-19, especially treatments for patients with severe or late-stage disease. Dexamethasone therapy was reported to reduce 28-day mortality in patients requiring supplemental oxygen compared with usual care (21.6% *vs* 24.6%; age-adjusted rate ratio, 0.83 [95% CI, 0.74-0.92]) (4), and remdesivir was reported to improve the time to recovery (hospital discharge or no supplemental oxygen required) from 15 to 11 days (5). In a randomized trial of 103 patients with COVID-19, convalescent plasma did not shorten the time to recovery (6). Ongoing trials are testing antiviral therapies, immune modulators, and anticoagulants [see review (7)]; however, there is no specific antiviral treatment recommended for COVID-19.

Chimeric antigen receptors (CARs) are synthetic receptors that redirect T cell activity towards specific targets (8). With the remarkable success of CAR-engineered T (CAR-T) cells for treating haematological malignancies, there is a rapid growing interest in developing other kind of CAR-engineered lymphocytes, such as CAR-NK for cancer therapy (9). A CAR construct includes antigen-recognition domains in the form of a single-chain variable fragment (scFv) or a binding receptor/ligand in the extracellular domains, a transmembrane domain providing the scaffold and signaling transduction, and intracellular domains from the T cell receptor (TCR) and costimulatory molecules that trigger T cell activation (10). Based on the longstanding interest in harnessing macrophages to combat tumor growth (11, 12), human macrophages engineered with CARs have been developed and characterized for their antitumor potential. Macrophages, critical effectors of the innate immune system, are responsible for sensing and responding to microbial threats and promoting tissue repair. We therefore hypothesize that CAR macrophages can be used to combat SARS-CoV-2. However, the hyperinflammatory macrophage response, which has been found to be damaging to the host, particularly in severe infections, including SARS-CoV-2, and cytokine release syndrome (CRS), which is also the most significant complication associated with CAR-T cell therapy, raise questions regarding the safety of using CAR macrophages for virus clearance.

In this report, we developed a series of chimeric antigen receptors based on recognition of the S protein and tested their ability to induce phagocytosis of SARS-CoV-2 virions. MER Proto-Oncogene Tyrosine Kinase (MERTK), which is a member of the Tyro-Axl-MerTK (TAM) family of protein, is highly expressed on macrophages and has a number of ligands, notably Gas6 and protein S, either as free proteins or attached to apoptotic cells during MERTK-mediated clearance of apoptotic cells (13). Interestingly, we reported that one CAR with the intracellular domain of MERTK did not show a notable killing effect in antigen-expressing cell-based models compared with other CARs but did demonstrate antigen-specific clearance of SARS-CoV-2 virions *in vitro* without the secretion of proinflammatory cytokines.

## Methods

### Cell Lines and Primary Human Cells

All cell lines were purchased from the American Type Culture Collection (ATCC; Manassas, VA). The identities of the cell lines were verified by STR analysis, and the cell lines were confirmed to be mycoplasma free. 293 and Vero cells were maintained in DMEM supplemented with 10% fetal bovine serum, and THP-1 cells were maintained in RPMI medium supplemented with 10% fetal bovine serum. Cell culture media and supplements were obtained from Life Technologies, Inc. For primary human cells, the peripheral blood samples were collected from healthy donors. After sample collection, PBMCs were isolated by density gradient centrifugation. To develop primary human macrophages, the monocytes were purified using anti-CD14 magnetic beads (Miltenyi Biotec, USA) and the purified monocytes were cultured in RPMI 1640 medium contains 10% Human serum and 0.05% Glutamine (Sigma, USA) for 7 days at 5% CO2 and 37°C. The primary human macrophages were identified by morphologic observation and flow cytometric analysis followed by anti-CD68 staining.

### Vector Construction

**Figure 1:**
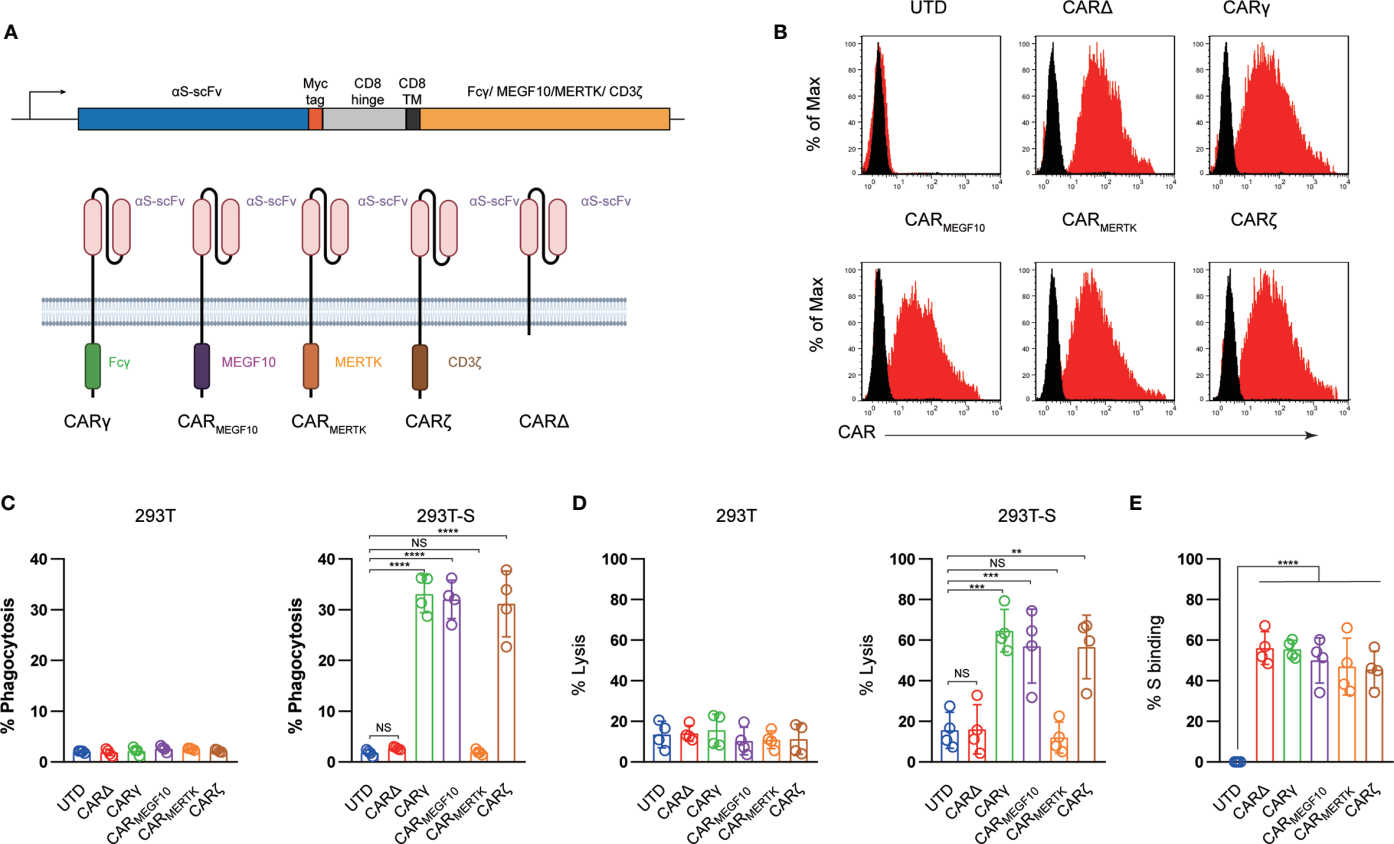
Generation and characterization of CAR macrophages. **(A)** Vector maps of tested CAR designs and schematics showing the structures of CARs used in the study. Figure created with BioRender. **(B)** Membrane-bound CAR expression. Forty-eight hours after retroviral transduction, the expression of synthetic receptors on THP-1 cells was detected by staining with an anti-MYC antibody, followed by flow cytometry analysis. Untransduced THP-1 cells were used as a negative control. The histograms shown in black correspond to the isotype controls, whereas the red histograms indicate positive fluorescence. **(C)** FACS-based phagocytosis of 293T cells or 293T-S target cells by UTD or different CAR macrophages. Statistical significance was calculated with one-way ANOVA with multiple comparisons. **(D)** Killing of 293T or 293T-S cells by UTD or anti-S CAR macrophages at 24 h assessed with a luciferase-based assay. **(E)** Flow cytometry analyses of CAR macrophages stained with a biotinylated S protein followed by streptavidin-FITC. The histograms shown in black correspond to the use of isotype controls with streptavidin-FITC, whereas the red histograms indicate positive fluorescence. The results shown represent three **(B)** independent experiments. Data are the shown as the mean ± s.d. of four independent biological replicates **(C–E)**. P values were derived by one-way ANOVA followed by Tukey’s posttest **(C–E)**. **p<0.01, ***p<0.001, ****p<0.0001. NS, Not Significant. The circles represent individual data.

The sequence encoding the scFv generated from CR3022 was chemically synthesized. As shown in **Figure 1A**, synthetic receptors contained the human CD8α signal peptide followed by the scFv linked in-frame to the hinge domain of the CD8α molecule, transmembrane region of the human CD8 molecule, and intracellular signaling domains of the FCER1G, MEGF10, MERTK or CD3ζ molecules. The cDNA sequences containing the various fusion constructs were cloned into a third-generation lentiviral vector in which the CMV promoter was replaced with the EF-1α promoter, the pELNS vector (14). High-titer replication-defective lentiviruses were produced and concentrated (14). Lentiviral infection was used to stably express CAR constructs in THP-1 cells.

### FACS-Based Phagocytosis Assay

UTD or CAR-expressing THP-1 cells were cocultured with GFP^+^ 293T cells or GFP^+^ 293T-S (S^+^) target cells for 4 h at 37°C. The effector-to-target (E:T) ratio was 1:1, and 1 × 10^5^ cells were used as both effector cells and target cells. After coculturing, the cells were harvested and stained with an anti-CD11b APC-Cy7-conjugated antibody (M1/70, BioLegend) and analyzed by FACS using a FACSCalibur flow cytometer (BD Biosciences). The percentage of phagocytosis was calculated based on the percent of GFP^+^ events within the CD11b^+^ population. Data are represented as the mean ± standard error of quadruplicate wells.

### Flow Cytometry

Cell-surface staining was performed for 45 min at 4°C and was analyzed using a FACSCalibur flow cytometer (BD Biosciences). A minimum of 1 × 10^4^ events per sample were examined.

### *In Vitro* Cytotoxicity Assay and Luciferase-Based Killing Assay

293T and 293T-S cells were used as targets in luciferase-based killing assays including control (UTD) or CAR macrophages. The effector-to-target (E:T) ratio was 10:1 for all the groups. Bioluminescence was measured using a Bio-Tek Synergy H1 microplate reader. The percent specific lysis was calculated on the basis of the experimental luciferase signal (total flux) relative to the signal of the target alone, using the following formula: %Specific Lysis = [(Sample signal —; Target alone signal)]/[Background signal — Target alone signal)] × 100.

### SARS-CoV-2 Pseudovirus and Cell Infection Experiments

The SARS-CoV-2 pseudovirus was constructed based on the spike genes of the strain Wuhan-Hu-1 (GenBank: MN908947) using published methods (15). The SARS-CoV-2 spike gene was chemically synthesized and cloned into a eukaryotic expression plasmid. 293T cells were first transfected with the S expression vector and then infected with a VSV pseudotyped virus (G∗ΔG-VSV), in which the VSV-G gene was substituted with luciferase expression cassettes. The culture supernatants were harvested and filtered at 24 h post infection. The SARS-CoV-2 pseudovirus could not be neutralized with anti-VSV-G antibodies, and no G∗ΔG-VSV was mixed with the SARS-CoV-2 pseudovirus stock. For cell-based infection assays, target cells were grown in plates until they reached 50%–75% confluency and then were inoculated with pseudotyped virus. The transduction efficiency was quantified at 16 h post transduction by measuring firefly luciferase activity according to the manufacturer’s instructions (Promega).

### Phagocytosis Assay

In all cases, SARS-CoV-2 S pseudotyped virions were pelleted (90 min at 14,000 rpm and 4°C), and after removal of the supernatant, the pellets were resuspended in RPMI medium and incubated with phagocytes (THP-1 cells or CAR macrophages) at 37°C for 1.5 h. After allowing time for phagocytosis, the cells were washed three times with PBS and incubated with Accutase (Innovative Cell Technologies) for 10 min at 37°C, followed by a final wash in Accutase. Intracellular staining for the S protein was performed for 60 min on ice after using a fixation/permeabilization kit (eBioscience) and then analyzed using a FACSCalibur flow cytometer (BD Biosciences). The phagocytic score was determined by gating the samples on events representing cells and was calculated as follows: Percent S protein positive × median fluorescence intensity (MFI).

### Cytokine Analysis

Cytokine analysis was performed on supernatants derived from cultures given the indicated treatments using a human cytokine 10-plex panel (Thermo Scientific) per the manufacturer’s instructions, with the panel results read on a Luminex Analyzer.

### Statistical Analysis

Unless otherwise specified, Student’s t test was used to evaluate the significance of differences between two groups, and ANOVA was used to evaluate differences among three or more groups. Differences between samples were considered statistically significant when P < 0.05.

## Results

To program engulfment based on recognition of the SARS-CoV-2 spike protein, we used a CAR design for the synthetic receptor strategy in our study. The synthetic receptors were constructed to contain an scFv derived from an antibody recognizing the virus spike protein, CR3022, which has been reported to bind with the receptor-binding domain of the SARS-CoV-2 S glycoprotein with high affinity, and the CD8 transmembrane domain present in the αCD19 CAR for T cells (12). For the cytoplasmic domains, we used the common γ subunit of Fc receptors (CARγ), MEGF10 (CAR_MEGF10_), MERTK (CAR_MERTK_) and CD3ζ (CARζ) in our study (**Figure 1A**). These cytoplasmic domains are capable of promoting phagocytosis by macrophages.

Next, we used lentiviral vector technology to express the fusion constructs in human macrophage THP-1 cells using clinically validated techniques (16). The cDNA sequences containing the various fusion constructs were cloned into a third-generation lentiviral vector in which the CMV promoter was replaced with the EF-1α promoter (17). An extracellular MYC epitope was cloned into the receptors to permit detection by flow cytometry. Lentiviral vector supernatants transduced THP-1 cells with high efficiency (**Figure 1B**). The phagocytic potential of human macrophage THP-1 cell lines expressing different CAR receptors or a truncated CAR receptor (CARΔ) lacking the intracellular domain was measured with a cell-based assay. Consistent with previous reports (11, 12), CAR macrophages and control untransduced (UTD) macrophages did not show notable phagocytosis of 293 cells; however, CAR_MEGF10_, CARγ and CARζ cells but not CAR_MERTK,_ CARΔ, or UTD macrophages phagocytosed Spike-bearing 293 cells in an S-specific manner (**Figure 1C**). CAR-mediated macrophage phagocytosis was further confirmed by a luciferase-based killing assay, and our data showed that CAR_MEGF10_, CARγ and CARζ cells eradicated S protein-expressing 293T cells in an antigen-specific manner (**Figure 1D**). Interestingly, CAR_MERTK_ and UTD macrophages showed-no difference in killing effect. Our data further showed that all synthetic receptors had the ability to bind the S protein (**Figure 1E**); therefore, the differences in phagocytosis and the lytic effect were not due to the affinity for the S protein. To further support the phagocytosis effect and cell killing effect of engineered macrophage is CAR dependent, CR3022 scFv were added to the culture to test the effect of CAR macrophages, our data showed when use anti-S scFv to block the interaction of S protein and CAR, the engineered macrophages showed no phagocytosis or killing effect, suggested that the observation of phagocytosis is dependent on the CAR receptor (**Figures S1A, B**).

**Figure 2:**
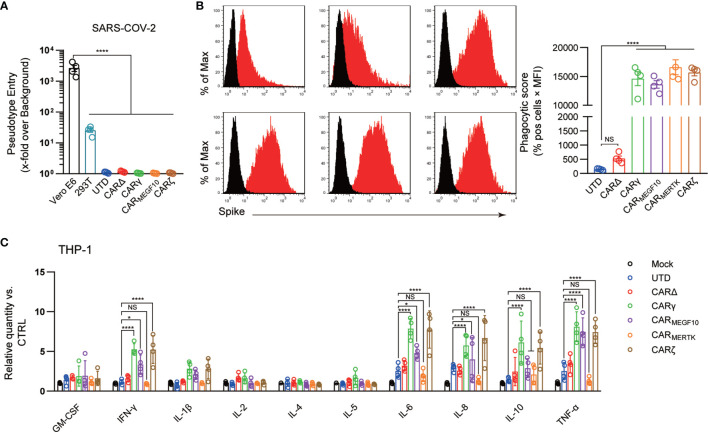
CARs mediate phagocytosis of SARS-CoV-2 virions. **(A)** Different cell lines were inoculated with a SARS-CoV-2 pseudotyped virus. At 16 h post inoculation, pseudotyped virus entry was analyzed by determining the luciferase activity in cell lysates. Signals obtained for particles bearing no envelope protein were used for normalization. The average of three independent experiments is shown. Error bars indicate the SEM. **(B)** The uptake of pseudotyped virions by UTD and CAR macrophages was analyzed by flow cytometry. Different cell lines were stained with an anti-S primary Ab. The histograms shown in black correspond to the isotype controls, whereas the red histograms indicate positive fluorescence. Data are reported as the phagocytic score (% positive cells x MFI, right panel). **(C)** Cell lines were infected with the SARS-CoV-2 pseudotyped virus or mock infected. Cytokine levels in the supernatants were determined by a multiplex bead array. The relative level was calculated as the ratio of the infected cells to the mock-infected THP-1 cells. Data are shown as the mean ± s.d. **(A–C)** of four independent biological replicates. P values were derived by one-way ANOVA followed by Tukey’s posttest (a–b) or two-way ANOVA followed by the Bonferroni posttest **(C)**; *p<0.05, ****p<0.0001. NS, Not Significant. The circles represent individual data.

Although there is currently no evidence that SARS-CoV-2 can infect THP-1 cells with or without IgGs (18), THP-1 cells have been shown to support antibody-mediated enhancement of SARS-CoV infection in previous studies (19). We therefore sought to determine whether synthetic receptors facilitate the entry of SARS-COV-2 into macrophages as host cells, as the extracellular domain of the CAR constructs has the capacity to directly bind to the S protein. Replication-defective VSV particles bearing coronavirus S proteins faithfully reflect key aspects of host cell entry by coronaviruses, including SARS-CoV-2 (20, 21). We therefore employed VSV pseudotypes bearing SARS-2-S to study the cell entry of SARS-CoV-2. Our data showed that Vero E6 cells were susceptible to entry driven by SARS-S (**Figure 2A**); however, no evidence of infection was detected in THP-1 cells with or without synthetic receptors.

Antibody-mediated phagocytosis and internalization of virions are important mechanisms of antiviral activity performed by macrophages against pathogens; however, using the phagocytosis assay developed for SARS-CoV-2, we observed low levels of phagocytic activity when UTD cells directly contacted virions. Phagocytic activity was not significantly increased when CARΔ cells rather than UTD macrophages were the phagocytes in the assay, suggesting that the extracellular domain of the CAR alone is not sufficient to induce strong virion internalization. CARγ, CAR_MEGF10_, and CARζ mediated similar significantly stronger levels of SARS-CoV-2 phagocytosis by THP-1 cells than CARΔ (**Figure 2B**). Unexpectedly, we also observed strong internalization of virions in CAR_MERTK_ cells, which did not show specific phagocytic or lytic effects on S protein-expressing 293T cells. Moreover, adding the anti-S scFv significant inhibited the virion internalization effect, supporting the role of CAR receptor in this process (**Figure S1C**). Since all the CARs exhibited the ability to induce phagocytosis of SARS-CoV-2 virions while there was no evidence of infection, these experiments strongly suggest the clearance of SARS-CoV-2 virions of CAR macrophages.

Because the systemic cytokine profiles observed in patients with severe COVID-19 show similarities to those observed in patients with macrophage activation syndrome, culture supernatants from THP-1 cells with different CARs treated with virions were further analyzed in a multiplex cytokine assay (**Figure 2C**). Following SARS-CoV-2 treatment of THP-1 cells, we observed slightly increased secretion of the cytokines IL-6, IL-8 and TNF-α, but no discernable patterns could be confidently drawn for GM-CSF, IL-1β, IL-2, IL-4, IL-5, IL-8, IL-10, and IFN-γ. CARΔ cells showed a cytokine profile similar to that of UTD macrophages. Notably, we observed not only strongly increased induction of IL-6, IL-8 and TNF-α but also induction of IFN-γ and IL-10 in SARS-CoV-2-treated CARγ and CARζ cells. However, for CAR_MERTK_ cells, we did not observe significant changes in cytokines.

**Figure 3:**
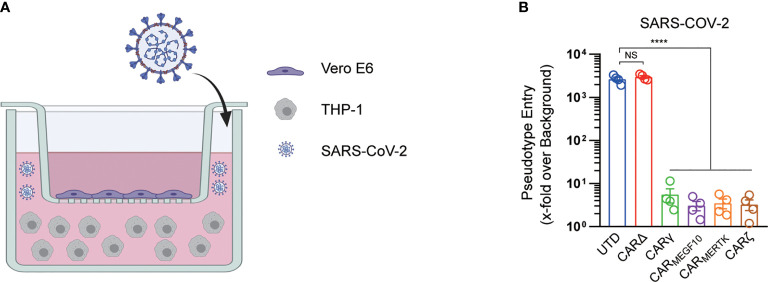
CARs mediate protection against SARS-CoV-2 infection. **(A)** The schematic shows the transwell coculture model. Figure created with BioRender. **(B)** Different cell cocultures were inoculated with the SARS-CoV-2 pseudotyped virus in the culture plate. At 16 h post inoculation, pseudotyped virus entry was analyzed by determining the luciferase activity in cell lysates of Vero cells. Signals obtained for particles bearing no envelope protein were used for normalization. Data are presented as the mean ± s.d. (a–c) of four independent biological replicates. P values were derived by one-way ANOVA followed by Tukey’s posttest. ****p<0.0001. NS, Not Significant. The circles represent individual data **(B)**.

We further used a transwell-based coculture model to evaluate the protective role of CAR macrophages in SARS-CoV-2 infection (**Figure 3A**). All the CAR-expressing macrophages potently inhibited Vero E6 cell infection with the SARS-CoV-2-S pseudotyped virus. Interestingly, CARΔ cells showed no protective effect in the infection assay, although they had a similar capacity to bind to the S protein, suggesting that the intracellular signaling domain is necessary for virion clearance by CAR macrophages (**Figure 3B**).

The above data demonstrated that CAR_MERTK_ cells can direct anti-virus phagocytic activity without induction of pro-inflammatory cytokines, we therefore sought to translate this platform to primary human macrophages. Primary human macrophages were generated from peripheral blood CD14+ monocytes and then engineered with MERTK CAR or CD3ζ CAR, termed as CAR_MERTK_-M cells or CARζ-M cells. Similar to the THP-1 cells, the resultant primary human CAR_MERTK_-M cells demonstrated no cell killing effect (**Figures S2A, B**) but a strong antigen-specific phagocytosis of SARS-COV-2 virions (**Figure S2C**), and this process did not induction of pro-inflammatory cytokines (**Figure S2D**). Moreover, adding anti-S scFv notably inhibit the phagocytosis effect, suggest that the biological effect of CAR_MERTK_-M cells is antigen dependent.

## Discussion

Macrophages, which protect against infections and scavenge the body’s worn-out or abnormal cells, are known for their phagocytic activity, antigen presentation capability, and flexible phenotype. The innate immune response of the pulmonary parenchyma, which is characterized by the differentiation of bone marrow-derived monocytes into macrophages, serves as a first-line defense against invading pathogens in the lungs (22). In general, monocytes/macrophages are able to remarkably limit viral replication. The monocyte-enhanced proinflammatory signaling molecule levels and antiviral responses provoked during viral infection have been shown for influenza, herpes, and Zika viruses (23). Moreover, it has recently been suggested that some COVID-19 patients have enhanced proinflammatory macrophage activity, which leads to accelerated production of inflammatory cytokines and chemokines and has mostly been observed in subjects with a poor prognosis (24).

To our knowledge, no synthetic cell-based immunotherapy has been investigated for COVID-19. CAR-expressing T cells have been demonstrated to be a very effective approach to treat B-cell cancer patients. Harnessing the power of engineered macrophages for the development of novel treatments for solid tumors is of great interest because CAR-T cell therapy is often hampered by the inability of T cells to penetrate solid tumors and the inhibitory tumor microenvironment (25). Consistent with a previous report (11), CAR receptors with cytosolic immunoreceptor tyrosine-based activation motifs (ITAMs) were capable of triggering specific engulfment and killing of antigen-expressing cells by macrophages. These CAR macrophages also showed strong phagocytosis of SARS-COV-2 virions in our data; however, this effect was accompanied by increased secretion of the proinflammatory cytokines IFN-γ, IL-6, and IL-8. In CAR-T cell therapy, engineered T cell expansion is usually accompanied by high-grade CRS with elevated circulating levels of interferon (IFN)-γ, granulocyte-colony stimulating factor (G-CSF), IL-6, IL-8, and IL-10. Recent reports have demonstrated that host-derived monocyte/macrophage and CAR-T cell interactions play an important role in CRS pathophysiology (26). This is of interest because increased serum levels of similar inflammatory cytokines (27–29) have been associated with COVID-19 severity and death. Interestingly, the secretion of IL-6, IL-8, TNF-α, IFN-γ and IL-10 was significantly elevated in CARγ and CARζ cells treated with SARS-CoV-2 virions, suggesting that these CAR macrophages may not be suitable for application in severe patients or patients with late-stage COVID-19.

Previous studies have shown that human immune cells, such as THP-1 cell lines, are susceptible to SARS-CoV infection (30). We did not observe any evidence that our SARS-CoV-2 pseudotyped virus infected THP-1 cells. Moreover, the uptake of virions by THP-1 cells was very low, even with a truncated CAR with the ability to bind to the S protein, suggesting that THP-1 cells did not innately engulf the virions. Notably, CAR_MERTK_, which was regarded as an unsuccessful receptor in a previous report (11) and showed no cellular killing effect on target cells when expressed in THP-1 cells in our assay, demonstrated a virion clearance capacity similar to that of CARγ and CARζ. Our data further support that CAR_MERTK_ mediates ‘immunologically silent’ virion removal, which does not elicit a proinflammatory response.

MER tyrosine kinase (MERTK), together with TRYO3 and AXL, belongs to the TAM family of receptor tyrosine kinases (RTKs). These receptors can be activated by a complex ligand consisting of phosphatidylserine (PtdSer) linked to the RTK by a vitamin K-dependent protein ligand, Gas6, or Protein S (31), playing a crucial role in innate immune cells. Gas6 has the capacity to bind all three receptors, while Protein S is a specific ligand of MERTK and TYRO3 (32). Apoptotic cells, exosomes, and cell debris are the main sources of the PtdSer component. In some cases, the PtdSer component is also provided by patches of exposed PtdSer on living cells (including T cells) (31). The activation of members of the TAM family of receptors generally induces an anti-inflammatory, homeostatic response in innate immune cells, diminishing excessive inflammation and autoimmune responses elicited by the ingestion of “self” (31). However, previous studies also proposed that enveloped viruses may hijack TAM receptors to facilitate attachment and infection *via* a PtdSer-dependent process termed “apoptotic mimicry” and act as potent TAM agonists, in turn inhibiting the type I IFN response in target cells (33). In our study, THP-1 cells expressing the synthetic receptor with the MERTK cytoplasmic domain were relatively resistant to virus infection but induced notable virion clearance. It should be noted that our study used very simple infection models; therefore, the assays lack numerous physiological and pathological factors, such as IgG or complement-mediated immune complexes, that may interfere with the behavior of engineered cells. Of cause, cells expressing synthetic receptors can be further engineered and developed to achieve precise control.

In summary, our data reveal that the CAR-based synthetic approach is applicable for COVID-19 treatment. In addition to direct virion clearance by CAR macrophages, we found evidence that MERTK-based CAR receptors did not induce further upregulation of proinflammatory cytokine levels, thereby raising the possibility that CAR macrophages may be useful as potent therapeutics in severe COVID-19.

## Data Availability Statement

The raw data supporting the conclusions of this article will be made available by the authors, without undue reservation.

## Author Contributions

WF, CL, KQ, ZM, TL, JZ, and SH designed and performed research. WF, CL, and SH analyzed the data. WF, CL, and SH wrote the paper. All authors contributed to the article and approved the submitted version.

## Funding

This study was supported by the National Natural Science Foundation of China (grant nos. 82041012, 81773261, 31970882, 81903140 and 81602690); the Shanghai Rising-Star Program (grant no. 19QA1411400); the Shanghai Sailing Program (19YF1438600); the Shanghai Biomedical Technology Support Project (20S11906600) and the Open Project Grant from Engineering Research Center of Cell & Therapeutic Antibody, Ministry of Education, of Shanghai JiaoTong University.

## Conflict of Interest

The authors declare the following competing interests: JZ is employed by company KOCHKOR Biotech, Inc. and is a shareholder at KOCHKOR Biotech, Inc. Shanghai. WF, JZ, and SH are inventors on intellectual property related to this work.

The remaining authors declare that the research was conducted in the absence of any commercial or financial relationships that could be construed as a potential conflict of interest.

## Publisher’s Note

All claims expressed in this article are solely those of the authors and do not necessarily represent those of their affiliated organizations, or those of the publisher, the editors and the reviewers. Any product that may be evaluated in this article, or claim that may be made by its manufacturer, is not guaranteed or endorsed by the publisher.
