# Supplementary material for: CAR Macrophages for SARS-CoV-2 Immunotherapy
Source: Front Immunol. 2021 Jul 23;12:669103. doi: 10.3389/fimmu.2021.669103 (PMC8343226; doi:10.3389/fimmu.2021.669103)
Supplement: Supplementary file 1 [file DataSheet_1.pdf]

Supplementary Information for:

CAR Macrophages for SARS-CoV-2 Immunotherapy

Contents **Page**

Supplementary Figures 2

Figure S1. Biological effect of engineered macrophages is dependent on CAR receptor. 2

Figure S2. Characterization of CAR engineered primary human macrophages. 3

Supplementary Figures

Figure S1

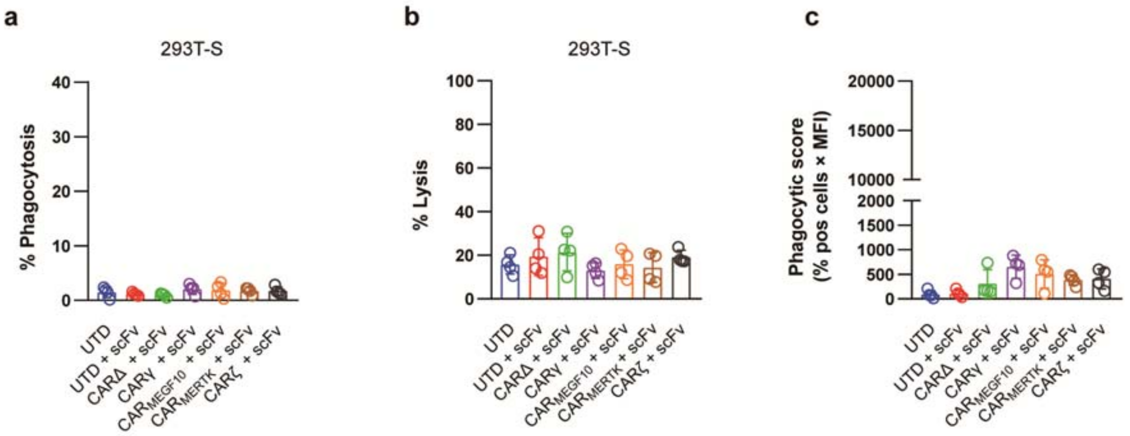

Figure S1. Biological effect of engineered macrophages is dependent on CAR receptor. a. FACS-based phagocytosis 293T-S target cells by UTD or different CAR macrophages with anti-S scFv. **b**. Killing of 293T-S cells by UTD or anti-S CAR macrophages with anti-S scFv 24 h assessed with a luciferase-based assay. **c,** The uptake of pseudotyped virions by UTD and CAR macrophages with scFv was analyzed by flow cytometry. The circles represent individual data.

Figure S2

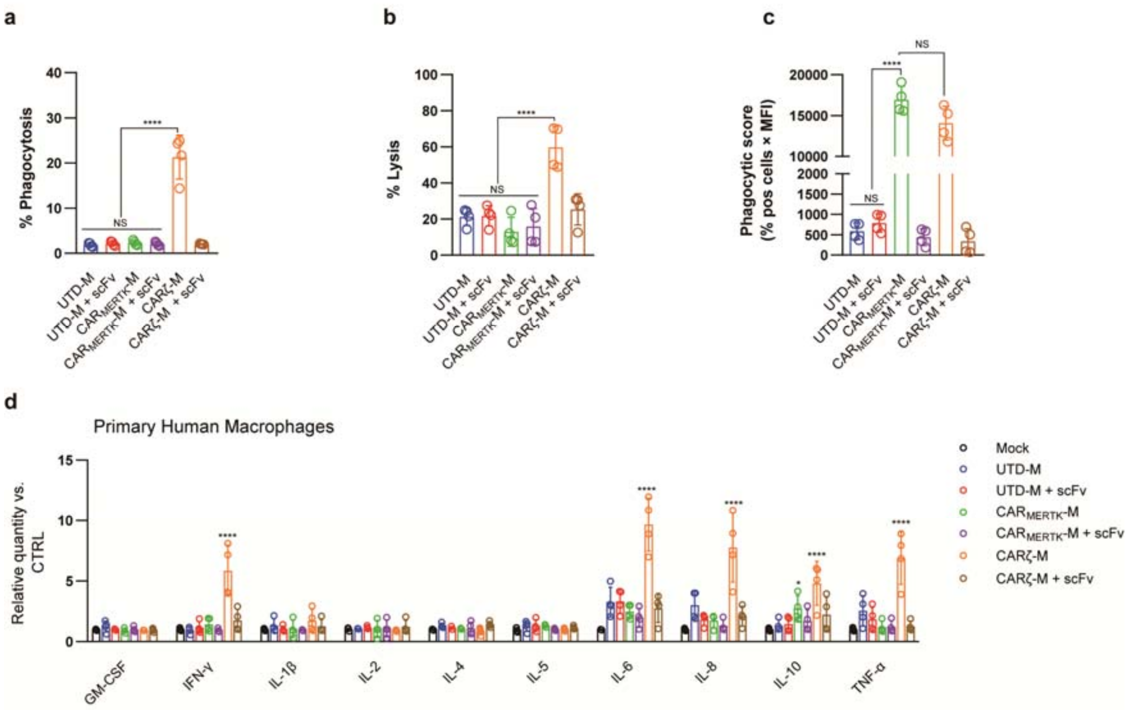

Figure S2. Characterization of CAR engineered primary human macrophages. a. FACS-based phagocytosis 293T-S target cells by different primary CAR macrophages with or without anti-S scFv. **b**. Killing of 293T-S cells by different primary CAR macrophages with or without anti-S scFv 24 h assessed with a luciferase-based assay. **c,** The uptake of pseudotyped virions by different primary CAR macrophages with or without scFv was analyzed by flow cytometry. **d,** different primary CAR macrophages were infected with the SARS-CoV-2 pseudotyped virus or mock infected. Cytokine levels in the supernatants were determined by a multiplex bead array. The relative level was calculated as the ratio of the infected cells to the mock-infected primary human macrophages. The circles represent individual data. P values were derived by one-way ANOVA followed by Tukey's posttest (a–c) or two-way ANOVA followed by the Bonferroni posttest (d); *p<0.05, **p<0.01, ***p<0.001, ****p<0.0001. The circles represent individual data.
